# SWPS3 – fast multi-threaded vectorized Smith-Waterman for IBM Cell/B.E. and ×86/SSE2

**DOI:** 10.1186/1756-0500-1-107

**Published:** 2008-10-29

**Authors:** Adam Szalkowski, Christian Ledergerber, Philipp Krähenbühl, Christophe Dessimoz

**Affiliations:** 1 Department of Computer Science, ETH Zürich, Zurich, Switzerland; 2 Swiss Institute of Bioinformatics, Lausanne, Switzerland

## Abstract

**Background:**

We present swps3, a vectorized implementation of the Smith-Waterman local alignment algorithm optimized for both the Cell/BE and ×86 architectures. The paper describes swps3 and compares its performances with several other implementations.

**Findings:**

Our benchmarking results show that swps3 is currently the fastest implementation of a vectorized Smith-Waterman on the Cell/BE, outperforming the only other known implementation by a factor of at least 4: on a Playstation 3, it achieves up to 8.0 billion cell-updates per second (GCUPS). Using the SSE2 instruction set, a quad-core Intel Pentium can reach 15.7 GCUPS. We also show that swps3 on this CPU is faster than a recent GPU implementation. Finally, we note that under some circumstances, alignments are computed at roughly the same speed as BLAST, a heuristic method.

**Conclusion:**

The Cell/BE can be a powerful platform to align biological sequences. Besides, the performance gap between exact and heuristic methods has almost disappeared, especially for long protein sequences.

## Background

Alignments are used in bioinformatics to compare biological sequences. The gold standard of sequence alignment is the optimal local sequence alignment with affine gap costs by Smith and Waterman [1,2]. Modern implementations achieve high performances through the use of SIMD instructions, which perform operations on multiple values in parallel. Such vectorized implementations for general purpose desktop processors include previous work by Wozniak [3], Rognes and Seeberg [4], and Farrar [5]. The latter is by a significant margin the fastest implementation on ×86 architectures with SSE2 (streaming SIMD extensions) instruction set. As for other platforms, Sachdeva *et al. *[6] ported the Altivec kernel of ssearch34 from the FASTA package [7,3] to the Cell/BE, but no implementation is publicly available, according to our knowledge. Another solution has been provided by Manavski and Valle [8] on general purpose graphics hardware.

In this article, we introduce swps3, an implementation of the Smith-Waterman algorithm that extends Farrar's work to the IBM Cell/BE platform. There, the improvement in runtime over results reported by Sachdeva *et al. *[6] are at least fourfold. The code also improves Farrar's work on ×86 architectures, mainly by supporting multi-core processors. In the following, we first present benchmarking results achieved with swps3 and compare them to other implementations. In the second part of the article, we discuss implementation details and the improvements over Farrar's algorithm.

## Results

By implementing Farrar's algorithm on IBM Cell/BE and exploiting all available processor cores, swps3 achieves higher alignment speed than previous implementations.

In the following, we compare swps3 with the following tools: ssearch35 [7], swsse [5], WU-BLAST 2.0 [9], and NCBI-BLAST 2.2.18 [10].

The queries consist of protein sequences aligned against release 55.1 of the Swiss-Prot [11] database featuring 129,199,355 amino acids in 359,942 sequences. The set of query sequences is an extension of Farrar's [5], with 7 longer sequences with length up to 4000 amino acids. Throughout the tests, we use the BLOSUM50 scoring matrix [12]. Curiously, the speed of NCBI-BLAST appears to be highly sensitive with respect to the scoring matrix. For instance, we observed runs that were twice as fast using BLOSUM62. All benchmarks were performed on Gentoo Linux with a 64-bit 2.6 kernel deployed on either an Intel Pentium Core 2 Quad Q6600 (2.4 GHz) or a Sony Playstation 3 featuring a Cell/BE (3.2 GHz) and 256 MiB XDRAM. Note that in this configuration, only six out of eight SPEs are available to the user.

**Figure 1:**
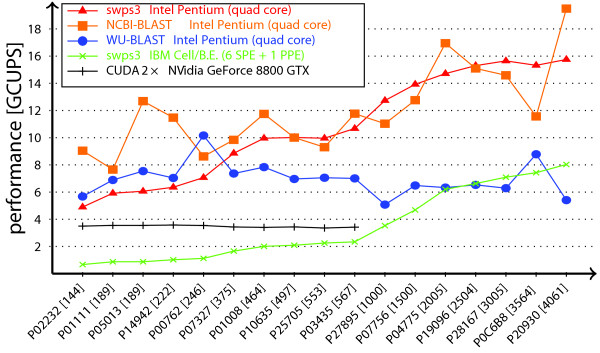
**Performance Evaluation**. Performance of gapped local alignment implementations on different multi-core architectures in GCUPS $\left(\frac{10^{9}\text{cell updates}}{s}\right)$.

Figure 1 presents the benchmarking results of our tool on different multi-core architectures. To put these results into a broader context, we included the runtime of multi-threaded WU-BLAST and NCBI-BLAST converted to a GCUPS-equivalent as well as performance data obtained by Manavski and Valle [8] on a GPU architecture. The runtime of ssearch and swsse are roughly the same as swps3, and are therefore omitted in the figure for the sake of clarity.

Throughout the benchmark, the Intel Pentium Q6600 was the fastest platform. On that machine, swps3 reaches an average performance of 10.7 GCUPS with a maximum of 15.7 GCUPS. The multi-threaded heuristic methods are the fastest with short query sequences, but for sequences longer than 300 amino acids, WU-BLAST is dominated by swps3. As for NCBI-BLAST, it exhibits roughly similar average performance as swps3. This suggests that to align long protein sequences, the use of heuristics is no longer justified.

Our implementation on Cell/BE performs relatively better with long sequence queries, when alignment scores are represented by 16-bit integers or 32-bit floating point values. In fact, it is able to catch up with the Intel Pentium Q6600 when processing (related) queries of 8000 amino acids (result not shown). On the other hand, the performances of the Cell/BE are limited by its sparse vector instruction set. For instance, the lack of support for 8-bit integer vectors restrains performance on short, unrelated sequences with low scores, which constitute the largest portion of the benchmark dataset.

## Implementation details

swps3 is largely based on previous implementations of the striped Smith-Waterman algorithm by [5]. This section describes our improvement, grouped by platform.

### Cell/BE specific improvements

The architecture of the Cell/BE differs in multiple aspects from other general purpose microprocessors. In addition to the main general-purpose PowerPC core (PPE), it features eight co-processors called *Synergistic Processing Elements *(SPE). The SPEs are not able to access main memory directly. Instead, each unit has a local storage of 256 kiB SRAM and a programmable DMA controller which performs bulk transfers between local storage and main memory, without interrupting program execution on the SPE. Large portions of our code are written as C++ templates and we apply intrinsics to access platform specific instructions. In doing so the compiler is responsible for optimizations. To ease this task, we manually unroll the inner loops, which results in a considerable performance gain. On ×86, execution time is reduced by early termination of inner loops, while on Cell/BE, early termination savings are offset by the overhead of evaluating conditional branch instructions. In this case also, static branch prediction fails to improve performance, and thus we moved code checking for conditions of early termination out of the inner loop. Although the memory consumption of the algorithm is linear in the length of the query and of the database sequence, memory is mainly consumed by the profile data. As improvement over Sachdeva *et al. *[6], we allow segmentation of the profile to support arbitrary query sequence lengths. Each segment is sequentially retrieved from main memory and processed. This requires storage of intermediate results: after processing the profile segment, we store the column gap scores (*F *in Farrar's notation) and maximum scores (*H*). Then, the next profile segment is fetched from main memory, replacing the old segment, and the alignment is resumed with the stored scores.

In experiments with long amino acid sequences (> 5000 residues), we tried double-buffering the profile (DMA transfer parallel to computation) but this resulted in worse performance. Indeed, pre-fetching requires additional local memory on the SPE, and thus decreases the length of the profile segments. Instead of pre-computing the profile on the PPE and transferring it to the SPE, we tried computing the profile directly on the SPE, thus only requiring the transfer of the query sequence and scoring matrix. Unfortunately, this approach resulted in a slightly higher execution time (results not shown). These results suggest that that the data transfers between PPE and SPE, even without double-buffering, only constitute a minor fraction of total execution time. Most biological query sequences are short enough for the entire profile to be loaded at once. The overhead of transmitting database sequences is also insignificant when considering that the Cell/BE is able to transmit 16 bytes every two processor cycles (25.6 GB/s at 3.2 GHz clock frequency).

### SSE2-specific improvements

While working on the code for the Cell/BE, we found a few minor aspects of Farrar's implementation that could be improved. By design, only unsigned 8-bit integers are stored in the scoring matrix. For an alignment with 16-bit scores, the profile is created by expanding these to 16-bit values by setting the upper bytes to zero. We were able to reduce the cache footprint caused by the profile by creating an 8-bit profile and using an unpacking operation (_mm_unpacklo_epi8) to extend it to 16-bit in the inner loop of the algorithm. This has shown especially beneficial for long query sequences. Altogether, our SSE2 implementation shows a very good cache efficiency. According to Valgrind [13], we have a L1 data miss rate of 0.8% and a L2 data miss rate of 0.087% when running P20930, the longest protein sequence in our test set, against Swiss-Prot.

Furthermore we restructured the *lazy F evaluation loop *(see [5]) by transforming it into two nested loops with specified index ranges to hint the compiler at execution counts. Also, the condition for early termination of this loop could be relaxed.

### Multi-threaded design

In order to exploit the whole potential of the Cell/BE, we designed a multi-threaded alignment algorithm to distribute the workload onto multiple SPEs and the PPE. After creating the profile, we fork one worker thread for each CPU core. It has proved most efficient to have six worker threads initializing a single SPE each and one thread performing an alignment using the Altivec instruction set of the PPE. The parent process handles file I/O and communicates with the worker threads over bidirectional pipes to supply them with database sequences and to collect alignment scores. Every worker thread computes the alignment of the query sequence with a separate database sequence. Note that the ×86 implementation also benefits from the multi-threaded design, as recent chips feature an increasing number of processing cores.

### Limitations

In the current version, swps3 is only able to compute scores of local protein sequence alignments with affine gaps. It does not display the resulting alignment. Users can select their own scoring matrix through the command line. Matrix entries are restricted to signed 8-bit integer values (i.e. from -128 to 127), but support for reading and scaling floating point matrices is available in the code. Sequences are limited to a maximal length of 10000 amino acids and scores are bounded by the relevant data type for computations (unsigned 16-bit integer by default).

## Conclusion

swps3 is a fast and flexible Smith-Waterman implementation for the Cell/BE, for PowerPC, and for ×86/×86_64 architectures. With a performance of up to 15.7 GCUPS on a quad-core Pentium and 8.0 GCUPS on the Sony Playstation 3, it is the fastest implementation we know of on both platforms. In addition, it also outperforms computation on general purpose graphics hardware as reported by Manavski and Valle [8], at significantly lower power consumption and cost.

## Availability

**project name**: SWPS3

**project website**: 

**operating systems**: Linux, Unix, Mac OS X

**programming language**: C, C++

**license**: MIT

## Competing interests

The authors declare that they have no competing interests.

## Authors' contributions

CD initiated and coordinated the project. AS, CL, PK contributed to the program code. AS, CD performed the analysis and drafted this manuscript. All authors read and approved the final manuscript.

## References

[B1] Smith TF, Waterman MS (1981). Identification of common molecular subsequences. Journal of Molecular Biology.

[B2] Gotoh O (1982). An improved algorithm for matching biological sequences. J Mol Biol.

[B3] Wozniak A (1997). Using video-oriented instructions to speed up sequence comparison. Computer Applications in the Biosciences.

[B4] Rognes T, Seeberg E (2000). Six-fold speed-up of Smith-Waterman sequence database searches using parallel processing on common microprocessors. Bioinformatics.

[B5] Farrar M (2007). Striped Smith-Waterman speeds database searches six times over other SIMD implementations. Bioinformatics.

[B6] Sachdeva V, Kistler M, Speight WE, Tzeng THK (2007). Exploring the Viability of the Cell Broadband Engine for Bioinformatics Applications. IPDPS.

[B7] Pearson, Lipman (1988). Improved Tools for Biological Sequence Comparison. Proc Natl Acad Sci.

[B8] Manavski SA, Valle G (2008). CUDA compatible GPU cards as efficient hardware accelerators for Smith-Waterman sequence alignment. http://www.biomedcentral.com/1471-2105/9/S2/S10.

[B9] Gish W (1996). WU-BLAST. Http://blast.wustl.edu.

[B10] Altschul SF, Madden TL, Schaffer AA, Zhang J, Zhang Z, Miller W, Lipman DJ (1997). Gapped BLAST and PSI-BLAST: a new generation of protein database search programs. Nucleic Acids Res.

[B11] Boeckmann B, Bairoch A, Apweiler R, Blatter MC, Estreicher A, Gasteiger E, Martin MJ, Michoud K, O'Donovan C, Phan I, Pilbout S, Schneider M (2003). The SWISS-PROT protein knowledgebase and its supplement TrEMBL in 2003. Nucleic Acids Res.

[B12] Henikoff S, Henikoff JG (1992). Amino acid substitution matrices from protein blocks. Proc Natl Acad Sci, USA.

[B13] Nethercote N (2004). Dynamic binary analysis and instrumentation. Tech Rep UCAM-CL-TR-606.

